# Reduced ocular perfusion after intravitreal Aflibercept and faricimab: an exploratory study for Tie2 receptor distribution in ophthalmic capillaries

**DOI:** 10.1007/s00417-025-06993-5

**Published:** 2025-10-13

**Authors:** Anna C. Schuhmayer, Nina A. M. Karl, Leon Pomberger, Markus Eidherr, Haidar Khalil, Martin Kallab, Clemens Strohmaier, Matthias Bolz, Anna Reisinger

**Affiliations:** 1 Medical Faculty, Johannes Kepler University, Linz, Austria; 2 Department of Ophthalmology, Johannes Kepler University, Linz, Austria; 3 Department of Ophthalmology, Kepler University Hospital, Krankenhausstraße 9, Linz, 4020 Austria

**Keywords:** Anti-VEGF, Aflibercept, Faricimab, Age-related macular degeneration, Laser speckle flowgraphy, Ocular perfusion

## Abstract

**Purpose:**

Intravitreal anti-vascular endothelial growth factor (anti-VEGF) injections reduce ocular perfusion in patients with neovascular age-related macular degeneration (nAMD). Faricimab blocks both, VEGF-A and Angiopoietin-2. The study investigated the effects of intravitreal Aflibercept or Faricimab on ocular perfusion in patients with nAMD.

**Methods:**

36 eyes of 36 Caucasian patients with nAMD were initially enrolled and treated with either Aflibercept (*n* = 18) or Faricimab (*n* = 18). Two patients were excluded after screening failures, resulting in 34 eyes (*n* = 17 per group) for analysis. Ocular perfusion was assessed using Laser Speckle Flowgraphy (LSFG) at baseline, and 1 and 4 weeks after the first injection. The main output parameter of LSFG, mean blur rate (MBR), was measured in the optic nerve head (ONH) and macula. MBR, defined by an ellipsoid region of interest (ROI), was calculated for the total ONH area (ONH-MA), major retinal vessels within the ONH (ONH-MV), and the tissue area containing microvasculature (ONH-MT). For macular measurements, a square ROI (150 × 150 pixels) was placed temporal to the optic disc to measure choriocapillaris perfusion (CHOR) without including main retinal vessels.

**Results:**

Faricimab group showed a significant decrease in MV (*p* = 0.006) after one week, while the decrease with Aflibercept was not significant after one week (*p* = 0.29). After 4 weeks, both groups showed a significant decrease (*p* = 0.003 and *p* = 0.017, respectively). For MT and CHOR, both groups showed a significant decrease in perfusion, both after one and after 4 weeks (*p* < 0.001).

**Conclusion:**

Faricimab caused a more rapid decrease in retinal perfusion, while choroidal perfusion was equally reduced by Aflibercept and Faricimab. These different responses in the vascular systems seem to indicate a different distribution of Tie2 receptors for Angiopoietin-2. These findings warrant further investigation into the role of Tie2 receptors in the vascular response to anti-VEGF therapies.

## Key messages

**What is known:**

• Intravitreal anti-VEGF agents reduce ocular perfusion in patients with nAMD.

**What is new:**

• This study examines Faricimab’s potential additional reduction in ocular perfusion due to dual-pathway inhibition compared to Aflibercept in nAMD patients.

• Faricimab induced a more rapid drop in retinal perfusion, while choroidal perfusion reacts comparable to Aflibercept and Faricimab.

• This supports the hypothesis of differential Tie2 pathway involvement, which requires further validation.

## Introduction

**Fig. 1:**
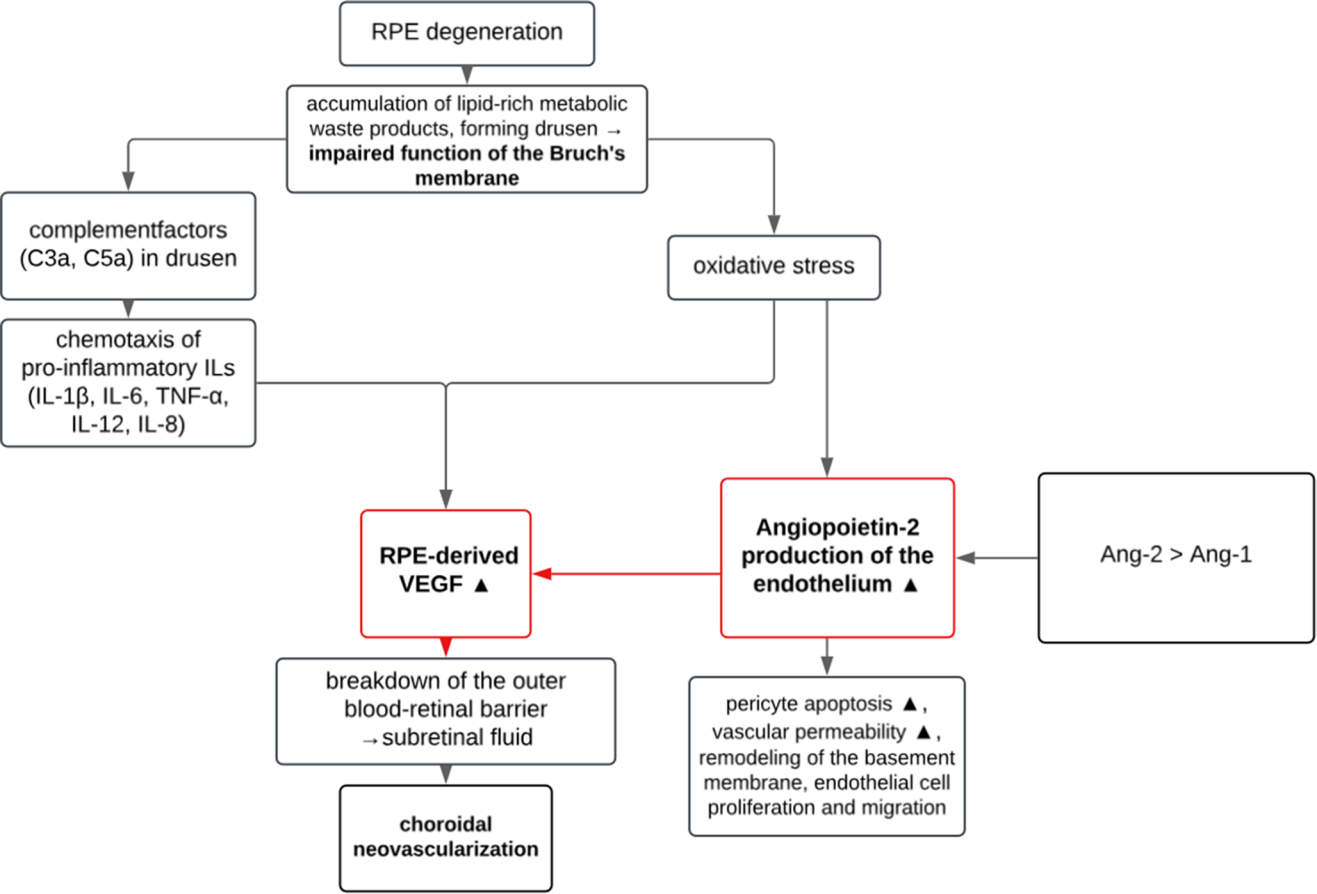
Schematic overview of the pathophysiology of neovascular AMD. Drusen formation, complement activation, proinflammatory cytokines and oxidative stress lead to an increased release of VEGF from the RPE and Ang-2 from endothelial cells. Inhibiting Tie2 signalling leads to Ang-2 destabilising vessels and increasing permeability. Together with VEGF, this results in disruption of the outer blood-retinal barrier and the development of CNV. RPE = retinal pigment epithelium; AMD = age-related macular degeneration; VEGF = vascular endothelial growth factor; Ang-2 = Angiopoietin-2; CNV = choroidal neovascularisation

VEGF is crucial for angiogenesis and increases vascular permeability by stimulating endothelial nitric oxide synthase (eNOS), leading to enhanced vasodilatation. Physiologically, in the eye VEGF is produced primarily by retinal pigment epithelial (RPE) cells as well as retinal ganglion cells, Müller cells, endothelial cells and pericytes. In nAMD drusen impair the function of the Bruch’s membrane and increased release of proinflammatory factors and oxidative stress trigger local overproduction of VEGF in the RPE, promoting choroidal neovascularization [1]. (see Fig. 1)

The Angiopoietin/Tie2 signaling pathway plays an essential role in maintaining vessel stability within the retinal vasculature [2]. Tie2 is a tyrosine kinase receptor, primarily expressed on endothelial cells (EC) of blood vessels [3, 4]. Angiopoietin-1 (Ang-1) and Angiopoietin-2 (Ang-2) are peptide ligands and bind Tie2 receptors. Under normal conditions, Ang-1 promotes stabilization of blood vessels and supports EC health by inhibiting Ang-2. However, under pathological conditions, Ang-2 production of the endothelium is increased and its binding to Tie2 receptors antagonizes the stability effect of Ang-1. This stimulates pericyte apoptosis and leads to increased vascular permeability, remodeling of the basement membrane, endothelial cell proliferation and migration, and stimulation of neovascularization [5]. In addition, the presence of Ang-2 potentiates the action of VEGF as well as enhancing endothelial sensitivity to VEGF [6]. 

Anti-VEGF therapy is the state-of-the-art treatment for nAMD. To date, there are various anti-VEGF agents available. Aflibercept, a commonly used anti-VEGF agent, neutralizes VEGF-A and PIGF. The specific structure of the Aflibercept molecule gives it an almost 100-fold higher binding affinity to VEGF-A than Ranibizumab and Bevacizumab [7]. Faricimab is a bispecific antibody that blocks both VEGF-A and Ang-2. Because of the interaction between VEGF and Ang-2, simultaneous inhibition of both signaling pathways could increase vessel stability and improve outcomes compared to current therapies [8]. 

Because of the multiple physiologic effects of VEGF, an impact on the ocular perfusion of anti-VEGF drugs seems likely. A reduction of retinal and choroidal perfusion in patients with nAMD after intravitreal Aflibercept has been shown before [9]. This could be relevant for the developing of geographical atrophy [10]. As for Faricimabs dual pathway inhibition, an additional effect on ocular perfusion is to be investigated in the current study.

## Materials and methods

### Patients

This prospective study included 36 eyes of 36 Caucasian adult patients with nAMD, treated with an intravitreal injection with either Aflibercept (Eylea^®^, Bayer, Germany) or Faricimab (VabysmoⓇ, Roche, Switzerland). Patients were recruited consecutively from the medical retina clinic of the Kepler University Hospital Linz, Austria. The study protocol was reviewed and approved by the local ethics committee (Ethikkommission des Landes Oberösterreichs; registration number 1269/2023) and followed the guidelines set forth in the Declaration of Helsinki [11]. Patients written informed consent was obtained before inclusion in the study.

The inclusion criteria were (1) age >50 years, (2) patients scheduled for an intravitreal injection with Aflibercept or Faricimab for treatment of exudative AMD in one eye and (3) a general good health condition with no uncontrolled systemic disease. The exclusion criteria included (1) ocular surgery (including intravitreal injection) during the 3 months preceding the study, (2) Ametropia >6 Dpt and (3) any relevant ophthalmic diseases or conditions potentially interfering with Laser Speckle Flowgraphy (LSFG) measurements (e.g. significant cataract, glaucoma, optic nerve head drusen, tilted disc, etc.). To minimize measurement inaccuracies, eyes with high refractive errors were excluded, as high myopia or hypermetropia can cause optical aberrations, altered ocular geometry, and choroidal thinning, all of which may compromise the reliability and comparability of LSFG perfusion measurements. Subjects were instructed to abstain from alcohol and stimulating beverages containing xanthine derivatives (e.g. tea, coffee) 12 h before the LSFG measurements [12]. Measurements were performed after resting for 10 min in a quiet, dark room with the subject in sitting position.

## Measurements

**Fig. 2:**
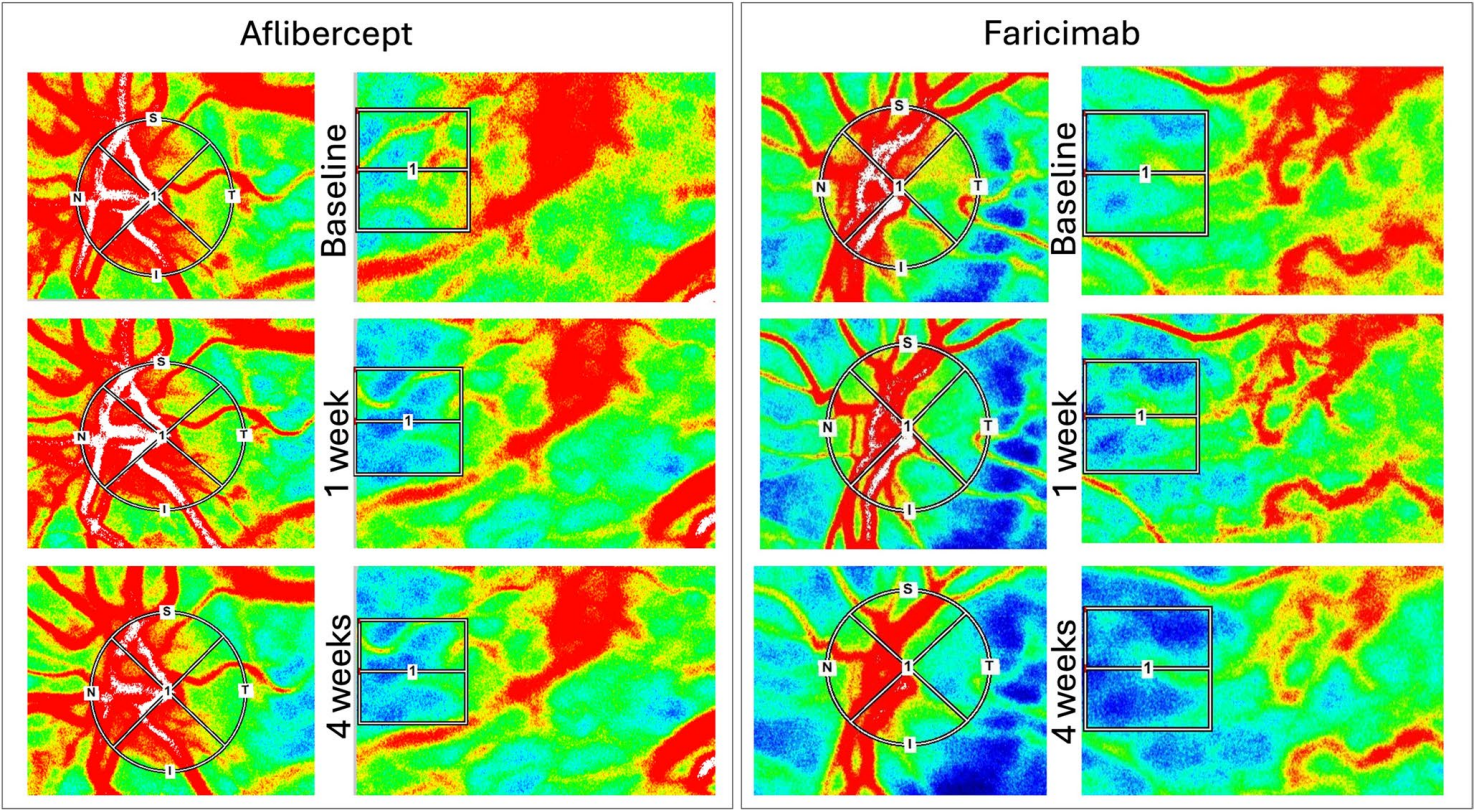
Representative colour map images from LSFG measurements of two patients from this study. Measurements centred at the ONH on the left side, measurements centred at the fovea on the right side. BL = baseline; 1 w = measurement 1 week after the first injection; 4 w, = measurement 4 weeks after the first injection. LSFG measurements of a patient after IVF (right); LSFG measurements of a patient after IVA (left)

Measurements were performed on the day of the scheduled intravitreal injection as well as after one and four weeks. Performed examinations included ETDRS best corrected visual acuity (BCVA) testing and Goldmann applanation tonometry. Optical coherence tomography (Spectralis, Heidelberg Engineering, Heidelberg, Germany) was employed to measure central retinal thickness (CRT). After pharmacological dilation of the pupil with 0.5% tropicamide eye drops (Mydriaticum Agepha Augentropfen; Agepha Ges.m.b.H., Vienna, Austria) patients were asked to rest for 10 min in a dark room before the systolic blood pressure (SBP) and diastolic blood pressure (DBP) were measured at the upper arm with a manometer in the sitting position. The mean arterial pressure (MAP) was calculated as MAP = DBP + 1/3 (SBP − DBP), and the ocular perfusion pressure (OPP) as OPP = 2/3 MAP − IOP. (see Table 1) Last, Laser Speckle Flowgraphy (LSFG) was performed with the RetFlow device (Nidek, Japan). The RetFlow consists of a fundus camera equipped with a diode laser at a wavelength of 830 nm and a digital charge-coupled device camera (750 × 360 pixels). A total of 118 images are acquired at a rate of 30 frames per second over a 4-second measurement period. The main output parameter of LSFG, mean blur rate (MBR), is calculated from the pattern of speckle contrast produced by the interference of a laser scattered by blood cells moving in the ocular fundus. MBR was calculated for the total ONH area, defined by an ellipsoid region of interest (ROI) (referred to as ONH-MA, “mean MBR of all area”). By using the on-board software, the MBR was calculated in the large retinal vessels within the ONH (ONH-MV, “mean MBR of vascular area”) and the tissue area containing the microvasculature (ONH-MT, “mean MBR of tissue area”). For analysis of the choriocapillaris (CHOR), a square ROI was set (150 × 150 pixels) in a temporal location one optic disc diameter away from the ONH, without including the main retinal vessels, as it was described before [13]. All ROI positions were saved and used for the follow up measurements. (shown in Fig. 2)

**Table 1:** Descriptive statistics and results of the t-test for testing of differences between the Aflibercept (A) and the faricimab (F) group at baseline. BCVA = best-corrected visual acuity; ETDRS = Early treatment diabetic retinopathy Study; MAP = mean arterial pressure; HR = heart ratio; OPP = ocular perfusion pressure; IOP = Intraocular pressure; CRT = Central retinal thickness

Variable	Aflibercept (A)/ Faricimab (F)	Mean ± SD	*p* (2-sided t-test)
Age [years]	A	77.8 ± 4.04	0.875
	F	77.5 ± 6.5	
BCVA [EDTRS letters]	A	67.9 ± 12.1	0.688
	F	69.4 ± 7.8	
MAP [mmHg]	A	118.1 ± 12.4	0.270
	F	113 ± 14.2	
HR [bpm]	A	65.5 ± 17.1	0.444
	F	69 ± 7.1	
OPP [mmHg]	A	68.9 ± 7.9	0.294
	F	65.7 ± 9.2	
IOP [mmHg	A	14.8 ± 2.7	0.651
	F	14.4 ± 2.5	
CRT [µm]	A	366.5 ± 103.6	0.900
	F	370.3 ± 68.4

## Intervention

After instillation of topical anesthetics (0.4% oxybuprocaine hydrochloride and lidocain; produced by the hospital pharmacy), sterilization of the eyelid (Betaisodona Lösung^®^, 11% povidone-iodine, Mundipharma, Limburg, Germany), and instillation of 1.25% povidone-iodine drops, 2.0 mg/0.05 mL of Aflibercept (Eylea^®^, Bayer, Germany) or 6.0 mg/0.05 mL of Faricimab (VabysmoⓇ, Roche, Switzerland) was injected into the vitreous cavity through a standard pars plana approach (3.5 mm posterior to the limbus) under sterile conditions.

## Statistics

### Sample size calculation

As no LSFG data for change in MA, MV and MT after intravitreal Faricimab were available at the time of study planning, a sample size calculation was performed based exclusively on data from Aflibercept [9]. A number of 14 subjects were found able to detect a statistically significant change over time. We considered a drop-out rate of 20% as only complete data sets were to be included in the statistical analysis, resulting in a planned total sample size of 18 subjects.

## Statistical analysis

Statistical analysis was performed with SPSS software version 24.0 (SPSS Inc., Chicago, IL, USA). Descriptive data are presented as mean and standard deviation. Normal distribution of the data in both groups was confirmed with the Shapiro-Wilk Test.

Change in the MBR after one and four weeks was calculated by the following formula:

$$\bigtriangleup MBR\;\left(MV,MT,MA\right)=MBR_{Follow-up}-MBR_{Baseline.}$$

Differences between the groups at baseline were tested for statistically significant differences with the Student’s t-test for unpaired data. Differences in change of BCVA, OPP and the LSFG parameters (MA, MV, MT, CHOR) between the groups were analysed with a General Linear Model (repeated measures of variances, rmANOVA, within and between subjects analysis). Data were tested for sphericity using the Mauchly’s test of sphericity, which was confirmed for all data. For post-hoc analyses, pairwise comparisons between baseline and week 1, and baseline and week 4 were conducted. The level of statistical significance was set to α = 0.05, however Bonferroni correction was applied to adjust for multiple comparisons within each parameter. The corrected level of significance is stated with the reported results, where it applies.

## Results

We included 18 eyes from 18 subjects in each group. One patient of each group was excluded prior to analysis due to a screening error, thus 17 eyes were analyzed per group. Prior anti-VEGF therapy was reported in 6 patients (35.3%) in the Aflibercept group and in 9 patients (52.9%) in the Faricimab group. Inclusion of pretreated patients required that no anti-VEGF therapy had been administered within the three months preceding study initiation.

At baseline, groups were comparable regarding age (Aflibercept 77.8 ± 4.0 vs. Faricimab 77.5 ± 6.5 years, *p* = 0.875, 95% CI for the difference: −5.2 to 5.8), BCVA (67.9 ± 12.1 vs. 69.4 ± 7.8 letters, *p* = 0.688, 95% CI: −6.1 to 9.5), MAP (118.1 ± 12.4 vs. 113 ± 14.2 mmHg, *p* = 0.270, 95% CI: −3.1 to 13.3), HR (65.5 ± 17.1 vs. 69 ± 7.1 bpm, *p* = 0.444, 95% CI: −8.2 to 15.8), OPP (68.9 ± 7.9 vs. 65.7 ± 9.2 mmHg, *p* = 0.294, 95% CI: −1.8 to 8.0), IOP (14.8 ± 2.7 vs. 14.4 ± 2.5 mmHg, *p* = 0.651, 95% CI: −1.4 to 2.2), and CRT (366.5 ± 103.6 vs. 370.3 ± 68.4 μm, *p* = 0.900, 95% CI: −55.3 to 62.1) (*p* > 0.05 for all parameters, unadjusted Student’s t-test).

Over time, OPP remained stable between visits in both groups (rmANOVA, Aflibercept *p* = 0.338, 95% CI for change: −2.5 to 5.0; Faricimab *p* = 0.135, 95% CI: −3.8 to 7.2, unadjusted). BCVA increased significantly from baseline to both follow-up visits in both groups (rmANOVA, *p* = 0.017, 95% CI for change: 1.2 to 8.9, unadjusted), with no significant difference between groups at any time point (*p* = 0.147, 95% CI: −2.5 to 10.4). Similarly, CRT decreased significantly from baseline to follow-up in both groups (*p* < 0.001, 95% CI: −45.0 to −18.3 Bonferroni-adjusted), with no significant difference between groups (*p* = 0.582, 95% CI: −12.1 to 18.3, unadjusted).

**Fig. 3:**
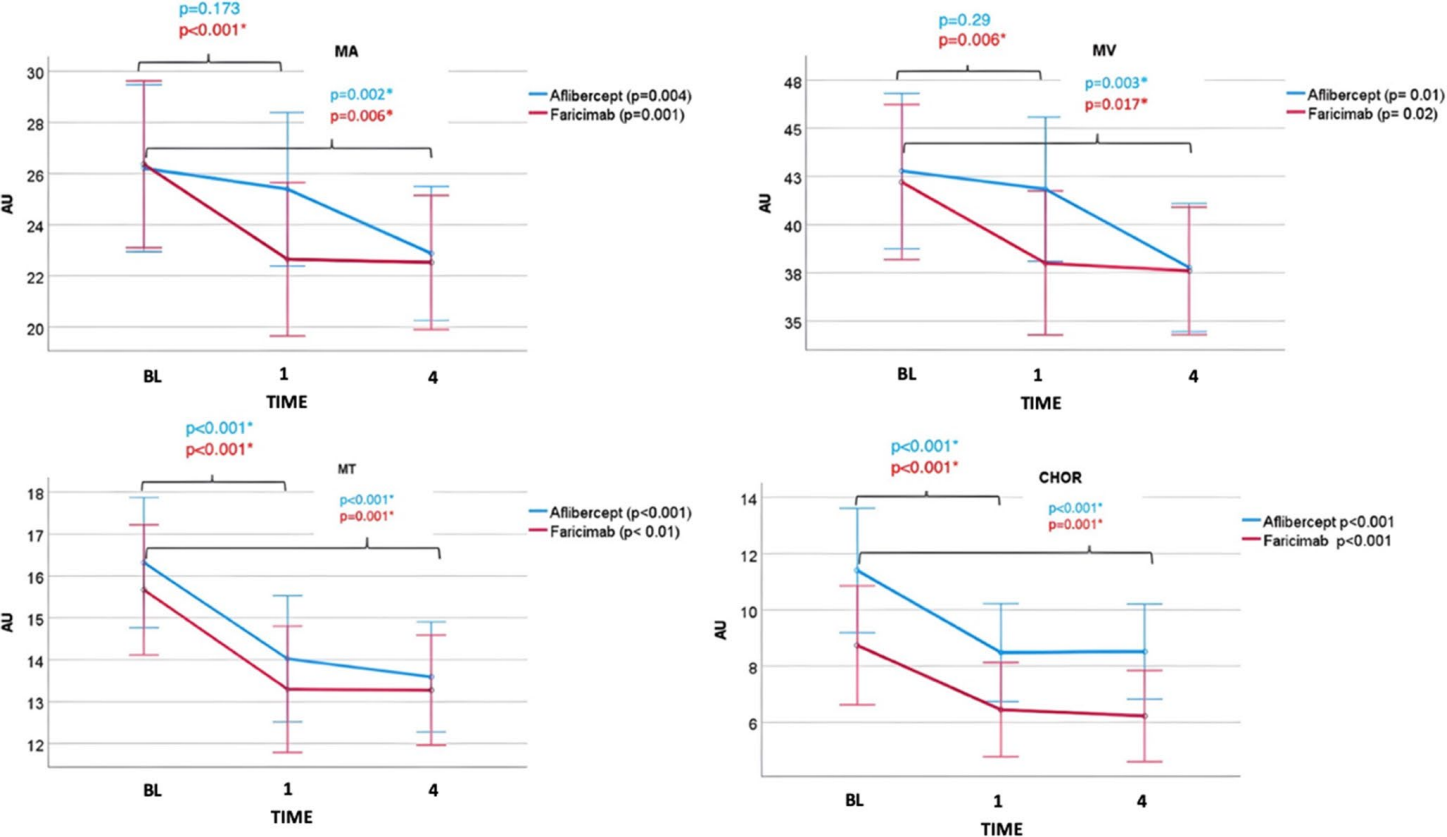
Line charts of mean and standard deviation (SD) indicating changes induced by intravitreal injection of Aflibercept (*n* = 17) or Faricimab (*n* = 17). AU = Arbitrary unit; ONH-MA = mean blur rate of the whole optic nerve head (ONH) region; ONH-MV = mean blur rate at region of big vessels within the ONH; ONH-MT = mean blur rate at region of microvasculature within the ONH; CHOR = mean blur rate of the choroid region of interest (ROI; BL = Baseline, 1 = 1 week after the first injection, 4 = 4 weeks after the first injection. Asterisks mark statistical significance after Bonferroni correction for multiple testing

A significant drop in MA, MV, and MT was observed from baseline to 1 and 4 weeks in both groups (rmANOVA across three time points, unadjusted). Time*group interaction showed no significant difference between groups (see Table 2). However, post-hoc t-test revealed a significant drop in MA and MV after one week only in the Faricimab group (MA *p* = 0.009; MV *p* = 0.006, both Bonferroni-adjusted α = 0.025). On the contrary, a significant drop in MT and CHOR after one week in both groups (*p* < 0.001, Bonferroni-adjusted α = 0.025) (see Fig. 3). Table 3<span class="EditNotAllowedNoToolTip oxe_aq_div oxe_aq" type="sps" id="1759375849929_1334862926681496" title="Missing citation for Table 3 was inserted here. Please check if appropriate. Otherwise, please provide citation for Table 3. Note that the order of main citations of tables in the text must be sequential." contenteditable="false">AQ</span>.

**Table 2:** Mean ± SD of mean blur rate (MBR) in whole region of interest at optic disc (MA), in retinal vessels (MV), ONH tissue perfusion (MT) and choroid (CHOR); V1-V3 = visit 1 to visit 3; tests for statistical significance of repeated measures ANOVA (rmANOVA) including mauchly’s test for sphericity and repeated measures ANOVA test for within-subjects interaction between time and group

	Aflibercept Mean ± SD	*p* (rmANOVA)	*p* (Mauchly test for sphericity)	Faricimab Mean ± SD	*p* (rmANOVA)	*p* (Mauchly test for sphericity)	*P* Time*group (Eta2)
MV V1	42.3 ± 7.3	0.041	0.67	42.2 ± 8.4	0.034	0.35	0.42 (0.027)
MV V2	40.8 ± 7.4			38.0 ± 8.1			
MV V3	37.9 ± 6.6			37.9 ± 6.7			
MT V1	16.2 ± 2.9	< 0.001	0.66	15.7 ± 3.2	< 0.001	0.26	0.77 (0.008)
MT V2	14.1 ± 2.8			13.3 ± 3.3			
MT V3	13.6 ± 1.8			13.4 ± 3.3			
MA V1	26.1 ± 5.7	0.01	0.38	26.4 ± 7.3	< 0.001	0.09	0.35 (0.032)
MA V2	24.3 ± 5.4			22.6 ± 6.5			
MA V3	23.0 ± 4.4			22.5 ± 6.0			
CHOR V1	11.4 ± 3.3	< 0.001	0.21	8.7 ± 3.7	< 0.001	0.07	0.54 (0.026)
CHOR V2	8.5 ± 2.6			6.4 ± 3.0			
CHOR V3	8.5 ± 2.6			6.2 ± 2.8

**Table 3:** Mean ± standard deviation (SD) of Δ mean blur rate (MBR) in whole region of interest at optic disc (MA), in retinal vessels (MV), ONH tissue perfusion (MT) and choroid (CHOR) calculated as ΔMBR (MV, MT, MA) = MBR_Follow−up_ - MBR_Baseline_; V1 = Baseline, V2 after 1 week, V3 after 4 weeks; CI confidence interval;

	Aflibercept Mean ± SD	95% CI	Faricimab Mean ± SD	95% CI
Δ MV V2-V1	−1.4 ± 6.8	−4.9/2.1	−4.2 ± 6.2	−7.3/−1.0
Δ MV V3-V1	−4.4 ± 6.3	−7.6/−1.2	−4.2 ± 8.4	−8.6/0.1
Δ MT V2-V1	−2.1 ± 1.9	−3.1/−1.1	−2.4 ± 1.9	−3.4/−1.4
Δ MT V3-V1	−2.6 ± 2.2	−3.8 ± −1.5	−2.3 ± 2.8	−3.7/−0.9
Δ MA V2-V1	−1.7 ± 3.1	−3.3/−0.1	−3.8 ± 3.8	−5.7/−1.8
Δ MA V3-V1	−3.1 ± 4.2	−5.2/−0.9	−3.9 ± 5.5	−6.7/−1.0
Δ CHOR V2-V1	−2.9 ± 1.6	−4.0/−1.9	−2.3 ± 1.5	−3.2/−1.4
Δ CHOR V3-V1	−2.9 ± 1.5	−3.9/−1.9	−2.5 ± 1.9	−3.7/−1.3

## Discussion

In this study, we investigated the effects of intravitreal Aflibercept or Faricimab on ocular perfusion in patients with nAMD by using LSFG. We conclude that Faricimab leads to a faster reduction in perfusion of the retinal vessels, but not in the optic nerve head microperfusion and the choroid, where effects of Aflibercept and Faricimab were comparable. This finding highlights the difference between the vascular compartments of the eye.

The retinal tissue is one of the most highly vascularized structures in the body. Its blood supply is derived from retinal and choroidal vessels. While the inner retinal layers are supplied by branches of the central retinal artery, the outer retinal layers are supplied via the choroid. The capillary lamina of the choroid consists of a dense plexus of fenestrated capillaries with diameters of 40–60 μm and is covered by pericytes in 11% [8, 14]. The retinal capillaries have a diameter of 5–15 μm and are covered by pericytes in 95% [8, 15]. It is already known, that fenestrated endothelial cells are especially dependent on VEGF [8, 16, 17]. 

One speculative explanation the pronounced drop of MV but not MT and CHOR in response to intravitreal Faricimab could be the presence of Tie2 receptors. These receptors have been shown in pericytes, and the greater number of pericytes on retinal capillaries compared to choroidal capillaries could explain that MV, but not MT or CHOR, reacts more pronounced to Faricimab than Aflibercept after one week [18]. Binding of Ang-2 to its receptors is enhanced in the presence of VEGF. Substantial VEGF binding after four weeks may therefore limit the efficacy of anti-Ang-2 therapy, resulting in a similar reduction in MV in both groups. The similarity of reduction in MT and CHOR suggests, that MT is formed at least in considerable part of the ONH perfusion supplied by the choroid.

While various studies, using different technologies, have shown the effects of anti-VEGF agents on ocular circulation in AMD [9, 19–22], this is the first trial investigating the differences between Aflibercept and Faricimab. In the past, a comparable drop in MV and CHOR has been shown for Brolucizumab and Aflibercept in patients with nAMD, thus the effects observed in the present study are likely to result from the additional blockage of Ang-2 in the Faricimab group rather than only different Anti-VEGF drugs [23]. 

In summary, the results of this study show that the induced drop in retinal perfusion is more pronounced after Faricimab treatment, while choroidal perfusion responds similarly to Aflibercept and Faricimab treatment. To our knowledge, this is the first study to directly compare the effects of intravitreal Aflibercept and Faricimab on ocular perfusion in patients with nAMD using LSFG, making this a novel approach. It provides functional insights beyond structural parameters and generates essential pilot data required for sample-size calculation in future confirmatory studies.

While these findings may suggest a potential role for Tie2 receptor distribution in the capillary layer of the choroid, this remains speculative. Vascular dynamics can be influenced by various factors beyond receptor density, and further research is needed to clarify the relationship between changes in perfusion and the involvement of the Tie2 receptor in the vascular response to anti-VEGF therapies. Several limitations of the present study should be acknowledged. The study was conceived as a pilot investigation, as no comparable data were available in the literature, and thus an exact sample size calculation could not be performed. This has to be kept in mind when interpreting the data. Consequently, the findings should be confirmed in a larger, adequately powered setting after performing a sample-size calculation with the provided data from this pilot study. Patients were not sub-classified according to nAMD subtype using either fluorescein or indocyanine green angiography. Potential sources of bias include selection bias due to the inclusion criteria and measurement bias, although LSFG measurements have been reported with high precision and reproducibility [24]. Systemic medication adherence between visits was not systematically monitored, which could potentially have influenced the observed outcomes, although ocular perfusion pressure remained stable throughout the observation period.

For future studies, we recommend conducting a priori power calculations based on our pilot data, implementing standardized protocols for assessing and documenting medication adherence, and performing repeated LSFG measurements to improve reliability and quantify measurement variability. Larger multicentre studies with longer follow-up periods are essential to validate and generalize the present findings.

## Declarations

This study was not supported by any sponsor or funder.
